# Basal cell carcinoma risk and solar UV exposure in occupationally relevant anatomic sites: do histological subtype, tumor localization and Fitzpatrick phototype play a role? A population-based case-control study

**DOI:** 10.1186/s12995-020-00279-8

**Published:** 2020-09-10

**Authors:** A. Bauer, E. Haufe, L. Heinrich, A. Seidler, H. J. Schulze, P. Elsner, H. Drexler, S. Letzel, S. M. John, M. Fartasch, T. Brüning, S. Dugas-Breit, M. Gina, W. Weistenhöfer, K. Bachmann, I. Bruhn, B. M. Lang, R. Brans, J. P. Allam, W. Grobe, S. Westerhausen, P. Knuschke, M. Wittlich, T. L. Diepgen, J. Schmitt, for the FB181 study group, Thomas Bieber, Sonja Bonness, Beate Brecht, Stephan Grabbe, Denise Küster, Linda Ruppert, Victoria Stephan, Anja Thielitz, Elisabeth Zimmermann

**Affiliations:** 1 Department of Dermatology, University AllergyCentre, Medical Faculty Carl Gustav Carus, Technical University Dresden, Fetscherstr 74, D-01307 Dresden, Germany; 2 Centre of Evidence-based Healthcare, University Hospital and Medical Faculty Carl Gustav Carus, Technical University Dresden, Dresden, Germany; 3 Institute and Policlinic for Occupational and Social Medicine, Faculty of Medicine Carl Gustav Carus, Dresden, Germany; 4 Institute and Outpatient Clinics of Occupational and Social Medicine, Medical Faculty Carl Gustav Carus, Technical University Dresden, Dresden, Germany; 5 Department of Dermatology, Dermatological Radiotherapy and Dermatohistopathology, Special Clinics Hornheide, Münster, Germany; 6 Department of Dermatology, University Hospital Jena, Jena, Germany; 7 Institute and Outpatient Clinic of Occupational, Social and Environmental Medicine, Friedrich-Alexander-University Erlangen-Nürnberg, Erlangen, Germany; 8 Institute of Occupational, Social and Environmental Medicine, University Medical Centre, Johannes-Gutenberg University Mainz, Mainz, Germany; 9 Department of Dermatology, Environmental Medicine and Health Theory, University of Osnabrück and Institute of Interdisciplinary Dermatological Prevention and Rehabilitation (iDerm) at the University of Osnabrück, Osnabrück, Germany; 10 Institute for Prevention and Occupational Medicine of the German Social Accident Insurance (DGUV), Institute of Ruhr University Bochum (IPA), Bochum, Germany; 11 Department of Clinical Social Medicine, Occupational and Environmental Dermatology, University of Heidelberg, Heidelberg, Germany; 12 Department of Dermatology, University Medical Centre, Johannes-Gutenberg University Mainz, Mainz, Germany; 13 Department of Dermatology and Allergology, Christine Kühne Center for Allergy Research and Education, University of Bonn, Bonn, Germany; 14 Department of Radiation, Institute of Occupational Health and Safety of the German Social Accident Insurance (DGUV), Sankt Augustin, Germany; 15 Department of Dermatology, Experimental Photobiology, Medical Faculty Carl Gustav Carus, Technical University Dresden, Dresden, Germany

**Keywords:** Occupation, Solar UV exposure, Basal cell carcinoma, Outdoor work, Histological subtype, Localization, Fitzpatrick phototype

## Abstract

**Background:**

A two-fold risk increase to develop basal cell carcinoma was seen in outdoor workers exposed to high solar UV radiation compared to controls. However, there is an ongoing discussion whether histopathological subtype, tumor localization and Fitzpatrick phototype may influence the risk estimates.

**Objectives:**

To evaluate the influence of histological subtype, tumor localization and Fitzpatrick phototype on the risk to develop basal cell carcinoma in highly UV-exposed cases and controls compared to those with moderate or low solar UV exposure.

**Methods:**

Six hundred forty-three participants suffering from incident basal cell carcinoma in commonly sun-exposed anatomic sites (capillitium, face, lip, neck, dorsum of the hands, forearms outside, décolleté) of a population-based, case-control, multicenter study performed from 2013 to 2015 in Germany were matched to controls without skin cancer. Multivariate logistic regression analysis was conducted stratified for histological subtype, phototype 1/2 and 3/4. Dose-response curves adjusted for age, age^2^, sex, phototype and non-occupational UV exposure were calculated.

**Results:**

Participants with high versus no (OR 2.08; 95% CI 1.24–3.50; *p* = 0.006) or versus moderate (OR 2.05; 95% CI 1.15–3.65; *p* = 0.015) occupational UV exposure showed a more than two-fold significantly increased risk to develop BCC in commonly UV-exposed body sites. Multivariate regression analysis did not show an influence of phototype or histological subtype on risk estimates. The restriction of the analysis to BCC cases in commonly sun-exposed body sites did not influence the risk estimates. The occupational UV dosage leading to a 2-fold increased basal cell carcinoma risk was 6126 standard erythema doses.

**Conclusion:**

The risk to develop basal cell carcinoma in highly occupationally UV-exposed skin was doubled consistently, independent of histological subtype, tumor localization and Fitzpatrick phototype.

## Background

Outdoor workers are exposed to many different hazards like solar ultraviolet (UV) radiation and heat, pesticides and other chemicals as well as urban air pollutants and allergens that may lead to health problems [1–4]. This article focuses on solar UV radiation.

Exposure to solar UV- radiation is the most important risk factor for the development of cutaneous basal cell carcinoma (BCC) [5]. Outdoor workers are exposed to significantly higher occupational UV exposure dosages than the rest of the population [6–8]. A recent meta-analysis of the epidemiological literature [9] (*n* = 23 studies) revealed a 43% increase in BCC risk in occupationally UV-exposed workers compared to indoor workers (OR 1.43; 95% CI 1.23–1.66; *p* = 0.0001). However, based on the available epidemiological evidence at this time, informed decision making to consider BCC related to occupational UV exposure as an occupational disease was not possible. This was due to the limited methodological quality of almost all included studies. Quality was low because of low numbers of included participants, poorly classified indoor and outdoor tasks and occupations, lack of quantification of occupational and leisure time UV exposure, lack of controlling for relevant confounders and high risk of bias [9]. A recent population-based case-control study [10] that included consecutive patients with incident BCC (*n* = 836) overcame limitations of former studies. Previous work from our group showed that individuals with high occupational solar UV exposure had a 1.84-fold significantly increased BCC risk (95% CI 1.19–2.83) compared with no occupational UV exposure and a 1.97-fold significantly increased BCC risk (95% CI 1.20–3.22) compared with moderate occupational UV exposure [10].

However, the study population of Schmitt et al. [10] consisted of a population-based sample of BCC patients with no special focus on occupational skin cancer. BCC were localized on all parts of the body. To analyze whether the occurrence of BCC is dependent on high occupational solar UV exposure in outdoor workers, further analyses were requested by the experts of the research monitoring group of FB-181 for commonly sun-exposed, occupationally relevant body sites (*n* = 643 out of 836 BCC in total), different histopathological subtypes and Fitzpatrick phototype [11, 12].

The aim of this study was to determine the extent to which the established significant association of occupational solar UV exposure and the occurrence of BCC varies depending on histological subtype, tumor localization and Fitzpatrick phototype. The analyses took the influence of age, age^2^, sex and non-occupational UV exposure into account.

The primary study questions were:

1. The risk to develop BCC at capillitium, face, lip, neck, dorsum of the hands, forearms outside or décolleté regarding high (≥ 90th percentile) versus moderate (44th–60th percentile) occupational UV exposure is influenced by histopathologic BCC subtype (nodular, sclerodermiform, superficial).
2. The risk to develop BCC at capillitium, face, lip, neck, dorsum of the hands, forearms outside or décolleté is influenced by tumor localization and Fitzpatrick phototype.
3. There is a positive dose-response relationship between cumulative solar UV exposure and the risk of developing BCC at capillitium, face, lip, neck, dorsum of the hands, forearms outside or décolleté.

## Methods

### Study design, setting, eligibility criteria for cases and controls

Six hundred forty-three cases suffering from incident BCC (first diagnosis of histologically confirmed BCC within the past 2 years, age ≥ 30 years) in commonly sun-exposed body sites (capillitium, face, lip, neck, dorsum of the hands, forearms outside, décolleté) out of 836 overall cases of a population-based, case-control, multicenter study performed from 2013 to 2015 in 8 German study centres [10] were included in the current post-hoc analysis. Cases with incident BCC in other body sites (*n* = 193), where occupational UV exposure is less likely, were excluded (upper arm, abdomen, back, buttocks, lower extremities). Sex and age matched controls (age ≥ 30 years) without a history of skin cancer were recruited from regional residents’ registration offices by mail. They were offered physical examination and expense allowance for study participation. In case of non-response, two reminders were sent out by mail. Individuals who responded (response rate 33.9%) to participate as potential controls received a standardized interview and full body dermatological examination by a trained dermatologist at the local recruitment site. Patients suffering from Xeroderma pigmentosum, Gorlin-Goltz-Syndrome or diseases with a genetic background that may result in BCC development were excluded.

### Interviews, clinical examinations, assessment of occupational and non-occupational UV exposure

Trained investigators (Residents and Consultant Dermatologists) performed standardized interviews, full body clinical examinations, including photo-damage scoring of 12 different body sites (capillitium, face, neck, back of the hand, upper arm inside, forearm and upper arm outside, décolleté, abdomen, back, buttocks, lower extremities), computer-assisted UV exposure assessments and non-occupational UV exposure assessments. Trained experts of the German Social Accident Insurance Institutions performed occupational UV-exposure assessments. Occupational and non-occupational UV exposure was assessed in detail. To reduce recall bias the participants were asked in the invitation letter to consider the major stages of their private life (e.g. childhood/school years, period of training, entry into working life, partnerships, family, possibly retirement from working life) as well as the duration of each period, place of residence, typical behavior in leisure time and on holidays, prior to the interview at the clinic. UV protection behavior was assessed in detail for leisure time. To detect occupational UV exposure and protection behavior, participants were asked about the major stages of their professional lives as well as the duration of and the activities in each period. Furthermore, they were asked at what times of the day and how long they usually spent time outdoors during their working life. Working periods abroad, e.g. on assembly work, and exposure to artificial UV radiation (e.g. during welding) should be remembered, too. Moreover, occupational UV exposure was assessed more precisely compared to previous studies by using insurance documents on the duration of outdoor occupations for the calculation of the cumulative occupational UV exposure. A detailed description of the calculation of the total non-occupational and occupational UV exposure was recently published elsewhere [10]. The investigators assessing occupational UV exposure were blinded concerning case or control status of the study participants. Leisure time UV exposure was validated by a blinded investigator using previously specified algorithms.

### Publication of study protocol

The study protocol was published in the project registry of the German Federal Ministry of Labour and Social Affairs (http://www.bmas.de/DE/Themen/Arbeitsschutz/Forschungsdatenbank/UVT/DGUV-FB_181_Hautkrebs_durch_UV-Strahlung_IFA4206.html) prior to the start of the study.

### Statistical analysis

Descriptive analyses were performed considering variable types and distributions. Conditional logistic regression analysis was used to estimate odds ratios (OR) and their 95% confidence intervals (CI) for BCC with total, occupational and non-occupational lifetime UV exposure. For the stratified analyses by phototype it was necessary to break the propensity-score matched pairs. All cases with BCC in sun-exposed body sites and with Fitzpatrick phototype 1/2 or 3/4 were compared to all controls with the respective Fitzpatrick phototype using logistic regression. Regression analyses were adjusted for sex, age, age^2^ and Fitzpatrick phototype. Regression analyses for occupational UV exposure were additionally adjusted for non-occupational UV exposure.

To investigate the dose-response relationship between lifetime total, occupational and non-occupational UV exposure (in SED) in commonly sun-exposed body sites and BCC risk, we first assessed empirical dose-risk relationships by calculating the OR and corresponding 95% CI for each exposure dosage by conditional logistic regression. Assuming a non-linear dose-response relationship [13], the empirical dose-response functions were fitted to the observed data using fractional polynomials in the linear regression models [14] aiming to maximize the R squared. Sample size calculation was performed with GPower 3.1 (Statistical Power Analyses for Windows, University of Düsseldorf). Assuming a statistical power of 80% and a probability of error of 5%, a total of at least 515 cases with BCC in commonly UV-exposed body sites must be included in the analyses for detecting a doubling of BCC risk for highly UV-exposed persons. SPSS 25 (IBM, Armonk, NY) and Stata 14 (Stata Corp, College Station, TX) were used for data analysis.

## Results

### Characteristics of the study population

Six hundred forty-three (81.6%) participants with incident BCC in commonly sun-exposed body sites (capillitium, face, lip, neck, dorsum of the hands, forearms outside, décolleté) out of 836 cases with incident BCC from the entire study population of Schmitt et al. [10], as well as matched controls were included in the analysis. Among cases, 40.4% had left school after 9 years compared to 27.8% among controls (Fisher’s exact test for education of cases versus controls: *p* < 0.001). Total occupational UV exposure was considerably higher in cases than controls (cases 1934.4 SED; controls 1317.6 SED; Wilcoxon rank-sum test: *p* = 0.0642). The difference in lifetime UV exposure was mainly attributed to differences in occupational exposure. No gender difference was seen, indicating successful matching (Table 1).

**Table 1:** Characteristics of study population

	study population		*P* values
	cases with BCC in occupationally UV-exposed body sites (*n* = 643)	controls (*n* = 643)	
**Sociodemographic characteristics**			
Age in years (mean ± SD, range)	66.3 ± 10.9 (29–89)	66.2 ± 9.7 (31–83)	not applicable (Propensity score matched)
Sex (n, %)			
Male	364 (56.6%)	368 (57.2%)	not applicable (Propensity score matched)
Female	279 (43.4%)	275 (42.8%)	
Education (n, %)			
No graduation	3 (0.5%)	1 (0.2%)	Fisher’s exact test: *p* < 0.001
9 years	260 (40.4%)	179 (27.8%)	
Middle school (10 years)	153 (23.8%)	141 (21.9%)	
High school (12 years)	172 (26.7%)	254 (39.5%)	
other	55 (8.6%)	68 (10.6%)	
**Clinical and anamnestic parameters**			
Fitzpatrick phototype (n, %)			
1	46 (7.2%)	31 (4.8%)	Pearson’s chi-squared test: *p* = 0.014
2	365 (56.8%)	338 (52.6%)	
3	223 (34.7%)	253 (39.3%)	
4	9 (1.4%)	19 (2.95%)	
5	0(0.00%)	2 (0.31%)	
Immuno-suppressant agents (intake), current and/or previous (n, %)			
yes	55 (8.6%)	38 (5.9%)	Pearson’s chi-squared test: *p* = 0.067
no	588 (91.4%)	605 (94.1%)	
Positive family history for skin cancer (n, %)			
yes	89 (13.8%)	61 (9.5%)	Pearson’s chi-squared test: *p* = 0.015
no	554 (86.2%)	582 (90.5%)	
Migration background (n, %)			
yes	35 (5.4%)	31 (4.8%)	Pearson’s chi-squared test: *p* = 0.613
no	608 (94.6%)	612 (95.2%)	
**UV exposure Standard Erythema Dose (SED; mean ± SD, range),**			
Total UV exposure	12,520.6 ± 4473.1 (4601.2 - 38,409.2)	12,002.2 ± 3976.1 (3980.9 - 30,984.9)	Wilcoxon rank-sum test: *p* = 0.0642
Occupational UV exposure (total)	1934.4 ± 3360.7 (0.0–22,763.6)	1317.6 ± 2439.1 (0.0–14,984.0)	Wilcoxon rank-sum test: *p* = 0.0182
Persons with occupational UV exposure > 0 SED	375 (58.3%)	357 (55.5%)	Pearson’s chi-squared test: *p* = 0.311
Occupational UV exposure for exposed (> 0 SED) persons	3316.9 ± 3845.8 (14.4–22,763.6)	2373.2 ± 2866.5 (3.8–14,984.0)	Wilcoxon rank-sum test: *p* = 0.0011
Non-occupational UV exposure (total)	10,586.2 ± 2708.2 (4460.3 - 20,988.9)	10,684.6 ± 2878.0 (3980.9 - 30,984.9)	Wilcoxon rank-sum test: *p* = 0.7421
Non-occupational UV exposure (vacation)	5541.0 ± 2025.5 (1566.7 - 16,122.0)	5654.3 ± 2170.1 (1663.6 - 14,992.0)	Wilcoxon rank-sum test: *p* = 0.6347
Non-occupational UV exposure (leisure time)	5052.2 ± 1373.8 (1624.7 - 13,338.0)	5032.2 ± 1387.8 (1235.1 - 17,459.9)	Wilcoxon rank-sum test: *p* = 0.5493

#### Histopathological subtype and tumor localization

Table 2 shows the distribution of histopathological subtypes of BCC in 643 male and female participants with BCC in commonly sun-exposed body sites. Nodular BCC (*n* = 403, 62.7%) was the most common histopathological subtype followed by sclerodermiform (*n* = 145, 22.6%) and superficial (*n* = 28, 4.4%) BCC (Table 2). No significant difference in distribution of these three BCC subtypes was seen between females and males (*n* = 576, *p* = 0.087).

**Table 2:** Distribution of all histopathologic BCC subtypes in males and females (*n* = 643)

Histopathologic subtype^**a**^	n (%)	Male	Female
Nodular	403 (62.7%)	230 (63.2%)	173 (62.0%)
Sclerodermiform	145 (22.6%)	69 (19.0%)	76 (27.2%)
Superficial	28 (4.4%)	18 (4.9%)	10 (3.6%)
Other (ulcus rodens, ulcus terebrans, pigmented)	31 (4.8%)	21 (5.8%)	10 (3.6%)
No data on subtype available	36 (5.6%)	26 (7.1%)	10 (3.6%)
Total	643	364	279

*n* number, *%* percentage

^**a**^ in commonly sun-exposed body sites

The mean age of tumor manifestation was slightly higher in superficial (68.3 ± 8.1 years of age) compared to nodular (66.1 ± 10.8 years of age) and sclerodermiform (66.0 ± 11.4 years of age) BCC.

Table 3 shows the distribution of histopathologic subtypes on distinct anatomical localizations in commonly sun-exposed body sites. Anatomical localizations differed significantly between the BCC subtypes (Pearson’s chi-squared test *p* < 0.001). Nodular and sclerodermiform BCC were mainly localized on the head (face, capillitium and lips). Superficial BCC predominated in the décolleté (Table 3).

**Table 3:** Anatomical distribution of BCC subtypes in commonly sun-exposed body sites (*n* = 643)*

Histopathologic subtype	Nodular	Sclerodermiform	Superficial	Other	No data available	All
Capillitium	29 (7.2%)	15 (10.3%)	0	2 (6.5%)	4 (11.1%)	50 (7.8%)
Face	335 (83.1%)	110 (75.9%)	9 (32.1%)	23 (74.2%)	19 (52.8%)	496 (77.1%)
Lip	3 (0.7%)	4 (2.8%)	1 (3.6%)	1 (3.2%)	1 (2.8%)	10 (1.6%)
Neck	12 (3.0%)	5 (3.5%)	1 (3.6%)	1 (3.2%)	2 (5.6%)	21 (3.3%)
Back of hand	2 (0.5%)	0	1 (3.6%)	0	1 (2.8%)	4 (0.6%)
Forearm outside	3 (0.7%)	2 (1.4%)	0	0	3 (8.3%)	8 (1.2%)
Décolleté	19 (4.7%)	9 (6.2%)	16 (57.1%)	4 (12.9%)	6 (16.7%)	54 (8.4%)
Total	403 (100%)	145 (100%)	28 (100%)	31 (100%)	36 (100%)	643 (100%)

* Pearson’s chi-squared test showed a significant association between body sites classified into head (capillitium/face/lip) versus the remaining body sites and BCC subtypes (nodular, sclerodermiform and superficial), *X*^2^ (2) = 75.3651, *p* < 0.001

#### Estimation of BCC risk

Participants with a high total UV exposure showed a 2-fold significantly increased risk to develop BCC in sun-exposed body sites versus low total UV exposure (OR 2.24; 95% CI 1.20–4.19; *p* = 0.012). The analyses for occupational UV exposure yielded significantly increased BCC risks for high versus no occupational UV exposure (OR 2.08; 95% CI 1.24–3.50; *p* = 0.006) and high versus moderate occupational UV exposure (OR 2.05; 95% CI 1.15–3.65; *p* = 0.015) (Table 4).

**Table 4:** Association of total and occupational UV exposure and risk for BCC in commonly sun-exposed body sites (*n* = 643 in each group)

UV exposure	Cases^**a**^	Contr.^**a**^	OR^**b**^	(95% - CI)^**b**^	p	OR^**c**^	(95% - CI)^**c**^	p
**Total UV exposure (leisure time and occupational exposure)**								
< 20th percentile (< 8985.0 SED)	115	143	Ref.	Ref.	Ref.	.	.	.
20. - < 40th percentile (8985.0 - 10,632.6 SED)	134	131	1.80	1.05–3.10	0.034	.	.	.
40th - < 60th percentile (10,632.7 - 12,565.6 SED)	138	132	1.70	1.01–2.84	0.044	.	.	.
60th - < 90th percentile (12,565.7 - 18,006.7 SED)	190	183	1.73	1.05–2.86	0.033	.	.	.
≥ 90th percentile (≥ 18,006.8 SED)	66	54	2.24	1.20–4.19	0.012	.	.	.
**Total UV exposure (high versus moderate exposure)**								
40th - < 60th percentile (10,632.7 - 12,565.6 SED)	138	132	Ref.	Ref.	Ref.	.	.	.
≥ 90th percentile (≥ 18,006.8 SED)	66	54	1.32	0.81–2.17	0.269	.	.	.
**Occupational UV exposure**								
< 44th percentile (< 2.9 SED)	268	286	Ref.	Ref.	Ref.	Ref.	Ref.	Ref.
44th - < 60th percentile (2.9–532.1 SED)	94	115	1.02	0.68–1.53	0.914	1.02	0.68–1.52	0.935
60th - < 90th percentile (532.2–5870.4 SED)	206	201	1.13	0.81–1.56	0.474	1.13	0.82–1.56	0.459
≥ 90th percentile (≥ 5870.5 SED)	75	41	2.08	1.24–3.48	0.006	2.08	1.24–3.50	0.006
**Occupational UV exposure (high versus moderate exposure)**								
44th - < 60th percentile (2.9–532.1 SED)	94	115	Ref.	Ref.	Ref.	Ref.	Ref.	Ref.
≥ 90th percentile (≥ 5870.5 SED)	75	41	2.03	1.14–3.61	0.016	2.05	1.15–3.65	0.015

^**a**^ number of cases and controls (contr)

^**b**^ adjusted for age, age^2^, sex, phototype

^**c**^ adjusted for age, age^2^, sex, phototype and non-occupational UV exposure

#### Estimation of BCC risk for different histopathological BCC subtypes

Participants with a high (≥ 90th percentile) total UV exposure showed a more than 2-fold significantly increased risk to develop nodular, sclerodermiform or superficial BCC in sun-exposed body sites (OR 2.27; 95% CI 1.17–4.42; *p* = 0.016) compared to participants with low total UV exposure. Similar results were observed for high versus low occupational UV exposure (OR 1.98; 95% CI 1.14–3.44; *p* = 0.015) and high versus moderate occupational UV exposure (OR 2.02; 95% CI 1.09–3.74; *p* = 0.026) (Table 5). There were no differences in the increased risk considering the histological subtypes. However, the number of cases was not sufficient to generate significant risk estimates (Tables S1-S3 supplementary material).

**Table 5:** Association of UV exposure and risk for BCC in cases with histopathological subtypes nodular, sclerodermiform or superficial and matched controls (*n* = 576 in each group)

UV exposure	Cases^**a**^	Contr.^**a**^	OR^**b**^	(95% - CI)^**b**^	p	OR^**c**^	(95% - CI)^**c**^	p
**Total UV exposure (leisure time and occupational exposure)**								
< 20th percentile (< 8985.0 SED)	107	132	Ref.	Ref.	Ref.	.	.	.
20. - < 40th percentile (8985.0 - 10,632.6 SED)	117	119	1.74	0.98–3.10	0.058	.	.	.
40th - < 60th percentile (10,632.7 - 12,565.6 SED)	125	118	1.71	0.99–2.94	0.053	.	.	.
60th - < 90th percentile (12,565.7 - 18,006.7 SED)	170	160	1.87	1.10–3.18	0.021	.	.	.
≥ 90th percentile (≥ 18,006.8 SED)	57	47	2.27	1.17–4.42	0.016	.	.	.
**Total UV exposure (high versus moderate exposure)**								
40th - < 60th percentile (10,632.7 - 12,565.6 SED)	125	118	Ref.	Ref.	Ref.	.	.	.
≥ 90th percentile (≥ 18,006..8 SED)	57	47	1.33	0.78–2.26	0.292	.	.	.
**Occupational UV exposure**								
< 44th percentile (< 2.9 SED)	251	264	Ref.	Ref.	Ref.	Ref.	Ref.	Ref.
44th - < 60th percentile (2.9–532.1 SED)	78	101	0.98	0.64–1.51	0.933	0.98	0.63–1.52	0.931
60th - < 90th percentile (532.2–5870.4 SED)	183	173	1.13	0.80–1.58	0.49	1.13	0.80–1.58	0.49
≥ 90th percentile (≥ 5870.5 SED)	64	38	1.98	1.14–3.44	0.015	1.98	1.14–3.44	0.015
**Occupational UV exposure (high versus moderate exposure)**								
44th - < 60th percentile (2.9–532.1 SED)	78	101	Ref.	Ref.	Ref.	Ref.	Ref.	Ref.
≥ 90th percentile (≥ 5870.5 SED)	64	38	2.02	1.09–3.74	0.026	2.02	1.09–3.75	0.026

^**a**^ number of cases and controls (contr)

^**b**^ adjusted for age, age^2^, sex, phototype

^**c**^ adjusted for age, age^2^, sex, phototype and non-occupational UV exposure

#### Estimation of BCC risk for different Fitzpatrick phototypes

Among other potential confounders (age, age^2^, sex), phototype was included in the risk estimation of BCC. For total UV exposure, the OR of phototype was 0.76 (95% CI 0.61–0.94; *p* = 0.012); for occupational UV exposure it was 0.79 (95% CI 0.64–0.98, *p* = 0.034). The stratified analyses of BCC risk (BCC in occupationally sun-exposed body sites, *n* = 643) by phototype showed no relevant differences of the risk estimates in participants with Fitzpatrick phototype 1/2 (n_cases_ = 411, n_controls_ = 491) and phototype 3/4 (n_cases_ = 232, n_controls_ = 342). The risk to develop BCC in commonly sun-exposed body sites was doubled for high versus no occupational UV exposure in phototype 1/2 (OR 1.73; 95% CI 1.05–2.85; *p* = 0.031) as well as phototype 3/4 (OR 2.13; 95% CI 1.12–4.05; *p* = 0.021). Similar results were obtained by comparing BCC risk for high versus moderate occupational UV exposure in phototype 1/2 (OR 2.11; 95% CI 1.21–3.68; *p* = 0.009) and phototype 3/4 (OR 1.88; 95% CI 0.94–3.75; *p* = 0.072; not statistically significant) (Tables 6 and 7).

**Table 6:** Association of total and occupational UV exposure and risk for BCC in phototypes 1/2 and matched controls (*n* = 451 in each group)

UV exposure	Cases^**a**^	Contr.^**a**^	OR^**b**^	(95% - CI)^**b**^	p	OR^**c**^	(95% - CI)^**c**^	p
**Total UV exposure (leisure time and occupational exposure)**								
< 20th percentile (< 8985.0 SED)	75	120	Ref.	Ref.	Ref.	.	.	.
20. - < 40th percentile (8985.0 - 10,632.6 SED)	90	102	1.71	1.10–2.66	0.018	.	.	.
40th - < 60th percentile (10,632.7 - 12,565.6 SED)	95	95	1.92	1.21–3.03	0.005	.	.	.
60th - < 90th percentile (12,565.7 - 18,006.7 SED)	111	136	1.60	1.02–2.51	0.042	.	.	.
≥ 90th percentile (≥ 18,006.8 SED)	40	38	2.12	1.17–3.85	0.013	.	.	.
**Total UV exposure (high versus moderate exposure)**								
40th - < 60th percentile (10,632.7 - 12,565.6 SED)	95	95	Ref.	Ref.	Ref.	.	.	.
≥ 90th percentile (≥ 18,006.8 SED)	40	38	1.11	0.64–1.90	0.714	.	.	.
**Occupational UV exposure**								
< 44th percentile (< 2.9 SED)	180	217	Ref.	Ref.	Ref.	Ref.	Ref.	Ref.
44th - < 60th percentile (2.9–532.1 SED)	54	80	0.82	0.55–1.24	0.353	0.82	0.55–1.24	0.353
60th - < 90th percentile (532.2–5.870.4 SED)	128	158	0.95	0.68–1.32	0.759	0.95	0.68–1.32	0.755
≥ 90th percentile (≥ 5870.5 SED)	49	36	1.73	1.05–2.85	0.031	1.73	1.05–2.85	0.031
**Occupational UV exposure (high versus moderate exposure)**								
44th - < 60th percentile (2.9–532.1 SED)	54	80	Ref.	Ref.	Ref.	Ref.	Ref.	Ref.
≥ 90th percentile (≥ 5870.5 SED)	49	36	2.11	1.21–3.68	0.009	2.11	1.21–3.68	0.009

^**a**^ number of cases and controls (contr)

^**b**^ adjusted for age, age^2^, sex, phototype

^**c**^ adjusted for age, age^2^, sex, phototype and non-occupational UV exposure

**Table 7:** Association of total and occupational UV exposure and risk for BCC in commonly sun-exposed body sites in phototypes 3/4 and matched controls (*n* = 287 in each group)

UV exposure	Cases^**a**^	Contr.^**a**^	OR^**b**^	(95% - CI)^**b**^	p	OR^**c**^	(95% - CI)^**c**^	p
**Total UV exposure (leisure time and occupational exposure)**								
< 20th percentile (< 8985.0 SED)	40	55	Ref.	Ref.	Ref.	.	.	.
20. - < 40th percentile (8985.0 - 10,632.6 SED)	44	66	1.11	0.61–2.04	0.724	.	.	.
40th - < 60th percentile (10,632.7 - 12,565.6 SED)	43	70	1.08	0.58–2.00	0.805	.	.	.
60th - < 90th percentile (12,565.7 - 18,006.7 SED)	79	117	1.25	0.69–2.24	0.459	.	.	.
≥ 90th percentile (≥ 18,006.8 SED)	26	34	1.58	0.75–3.33	0.228	.	.	.
**Total UV exposure (high versus moderate exposure)**								
40th - < 60th percentile (10,632.7 - 12,565.6 SED)	43	70	Ref.	Ref.	Ref.	.	.	.
≥ 90th percentile (≥ 18,006.8 SED)	26	34	1.46	0.76–2.81	0.254	.	.	.
**Occupational UV exposure**								
< 44th percentile (< 2.9 SED)	88	146	Ref.	Ref.	Ref.	Ref.	Ref.	Ref.
44th - < 60th percentile (2.9–532.1 SED)	40	63	1.14	0.69–1.86	0.61	1.13	0.69–1.86	0.618
60th - < 90th percentile (532.2–5.870.4 SED)	78	107	1.39	0.92–2.10	0.123	1.39	0.92–2.10	0.122
≥ 90th percentile (≥ 5870.5 SED)	26	26	2.13	1.12–4.05	0.021	2.13	1.12–4.06	0.021
**Occupational UV exposure (high versus moderate exposure)**								
44th - < 60th percentile (2.9–532.1 SED)	40	63	Ref.	Ref.	Ref.	Ref.	Ref.	Ref.
≥ 90th percentile (≥ 5870.5 SED)	26	26	1.87	0.94–3.73	0.074	1.88	0.94–3.75	0.072

^**a**^ number of cases and controls (contr)

^**b**^ adjusted for age, age^2^, sex, phototype

^**c**^ adjusted for age, age^2^, sex, phototype and non-occupational UV exposure

#### Occupations under risk

Outdoor occupations under risk in the highly occupationally UV-exposed group (≥ 5870.5 SED) included agriculture, animal and plant production, building and construction work, outdoor metal work, road construction and civil engineering, occupations in transport, street and vehicle cleaning as well as security staff and engineers with preponderant outdoor tasks.

#### Dose-response relationship between UV exposure and BCC risk

**Fig. 1:**
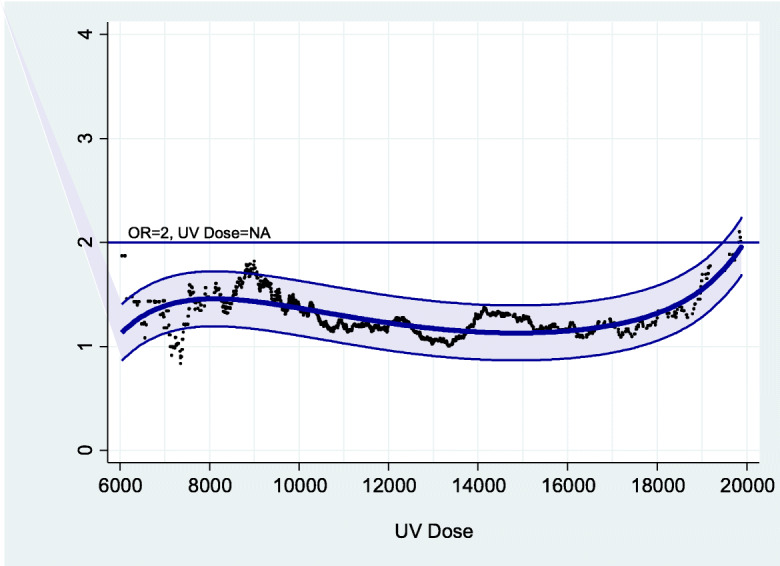
Empirical odds ratios. Dose-response curves with 95%-confidence band: dose-response relationship for *total* UV exposure. Odds ratios adjusted for sex, age, age^2^ and Fitzpatrick phototype using conditional logistic regression model (best fit): OR = 8.47E-9 * dose^2^–125,261,133 * 1/dose^2^–4.93 * LOG (dose) + 1.25E-9 * EXP (dose) + 47.13 (corrected R^2^ = 0.481). NA = not applicable

**Fig. 2:**
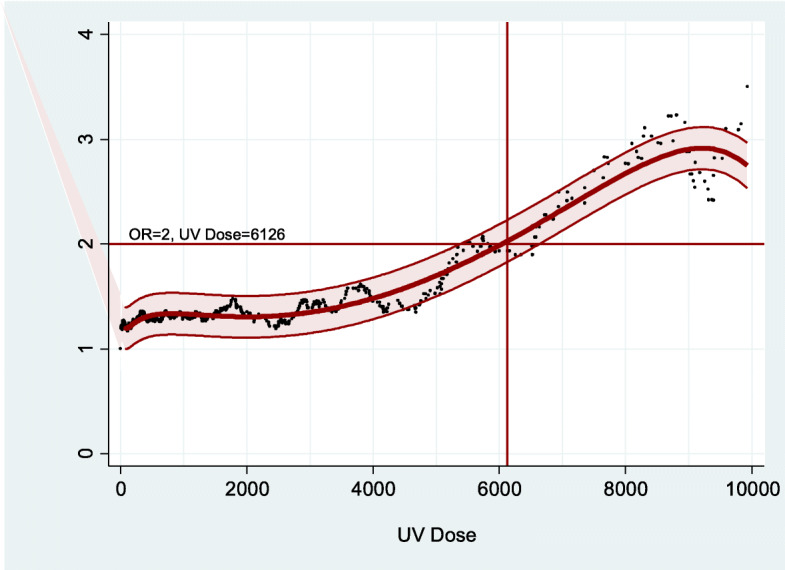
Empirical odds ratios. Dose-response curves with 95%-confidence band: dose-response relationship for *occupational* UV exposure. Legend: Odds ratios adjusted for sex, age, age squared, Fitzpatrick phototype and private UV exposure using conditional logistic regression model (best fit): OR = − 0.10 * SQRT (dose) - 58.76 * 1/dose + 66.51 * 1/dose^2^–37.35 * 1/SQRT (dose) + 6.32E-6 * 1/(dose * SQRT (dose)) + 1.81 * LOG (dose) - 5.52E-5 * EXP (dose) - 9.28 (corrected R^2^ = 0.944)

**Fig. 3:**
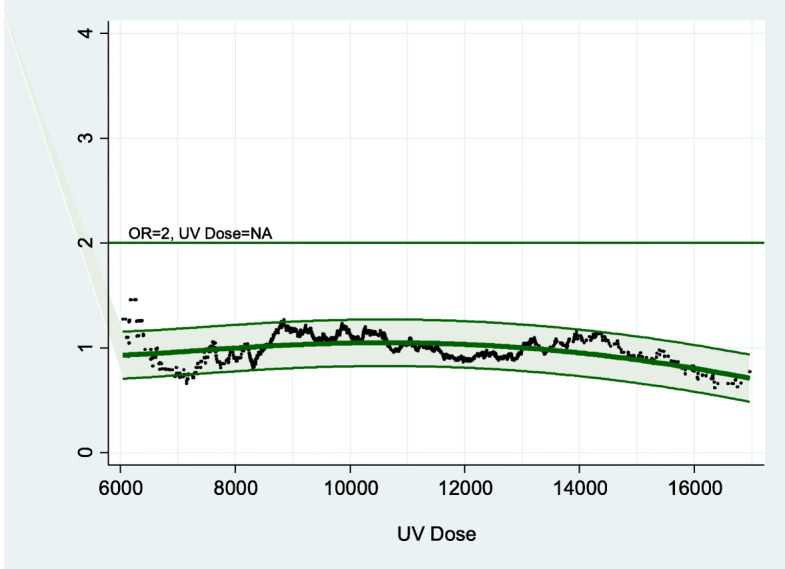
Empirical odds ratios. Dose-response curves with 95%-confidence band: dose-response relationship for *private* UV exposure. Legend: Odds ratios adjusted for sex, age, age squared and Fitzpatrick phototype using conditional logistic regression model (best fit): OR = − 5.73E-9 * dose^2^ + 20,282,607 * 1/dose^2^ + 1.65 * LOG (dose) - 13.78 (corrected R^2^ = 0.199). NA = not applicable

Adjusted dose-response curves for cases with BCC in commonly sun-exposed body sites and propensity-score matched controls (*n* = 643 in each group) were estimated for total, occupational and non-occupational UV exposure (Figs. 1, 2 and 3). Positive dose-response curves were observed especially for occupational UV exposure, with a doubling dose of 6126 SED for the risk to develop BCC in commonly sun-exposed body sites (Fig. 2).

## Discussion

In the present study, we demonstrated that BCC risk estimates in persons with high occupational UV exposure in commonly sun-exposed body sites were doubled consistently, irrespective of histological subtype, tumor localization and Fitzpatrick phototype. This analysis allowed us to substantiate the results of the recently published population-based, case-control study in patients with incident BCC [10]. The previous analysis yielded a doubled risk to develop BCC in persons with high compared to low or moderate occupational solar UV exposure but included BCC not localized in commonly sun-exposed body sites [10].

### Association of histopathological BCC subtype and risk estimates

In our data set the majority of nodular and sclerodermiform BCC were found on chronically sun-exposed body areas like head and neck. Superficial BCC were more often diagnosed on the trunk, where exposition to high intermittent UV exposures is more likely. In the literature comparable distribution patterns of histological subtypes are published [15–21]. Different susceptibilities to different sun exposure patterns and amounts have been discussed as possible reasons for the different distribution of histological subtypes on head and neck compared to trunk [12, 15–21].

To evaluate whether the different histological subtypes are differently related to occupational and non-occupational UV exposure, we performed multivariate regression analyses, adjusted for age, age^2^, sex and phototype. We found a consistently doubled risk to develop BCC in commonly sun-exposed body sites (capillitium, face, lip, neck, dorsum of the hands, forearms, décolleté) in participants with high versus low and moderate occupational UV exposure, respectively. We did not see any differences in the risk estimates in different histological subtypes for high cumulative UV exposure. Pelucchi et al. [12] showed a significantly increased risk for nodular (OR 1.53; 95% CI 1.08–2.18) but not for superficial (OR 0.71; 95%CI 0.44–1.15) BCC in patients exposed to occupational solar UV radiation. However, like in our study, subgroup analysis in cases with long duration occupational UV exposure revealed no significant risk increase for nodular BCC [12]. This indicates, if a certain threshold of occupational UV exposure is exceeded, the histological BCC subtype is irrelevant.

### Association of Fitzpatrick phototype and BCC risk

Concerning the association of Fitzpatrick phototype and BCC risk our study indicates that individuals with phototype 3/4 are at lower risk compared to individuals with phototype 1/2 for UV exposure in general as well as for occupational UV exposure. Importantly, high occupational UV-exposure was as harmful for individuals with phototype 1/2 as for those with phototype 3/4 leading to an approximately 2-fold increased risk for BCC on commonly sun-exposed body sites. This is confirmed by reports in the literature, where not only poor tanners with Fitzpatrick phototype 1 and 2 but also good tanners with darker phototypes were shown to be at substantial risk to develop BCC when highly UV exposed [13, 22–25]. However, reported risk estimates varied from no risk increase up to more than 6-fold risk increase for BCC development in light phototypes compared to darker phototypes [22–32]. These diverging results are probably due to different study designs, patient populations included (Northern/South Europa, South America, Australia), latitude, determination of phototype (by measurements, physician- or patient-reported) and statistical methods used. Based on the evidence in the literature and on our data we recommend that prevention strategies must address all outdoor workers regardless of phototype.

### Occupations under risk

Among BCC cases, we observed a significantly lower school education. A previous study found that outdoor work is associated with lower education [33]. This must be borne in mind when developing target group-specific prevention strategies.

### Dose-response relationship between occupational and non-occupational UV exposure and BCC risk

In our study population, we quantified the association of cumulative UV exposure and BCC risk for occupational, non-occupational and total UV exposure. The dose-response curve for occupational UV exposure, controlled for sex, age, age^2^, Fitzpatrick phototype, and non-occupational UV exposure showed a positive dose-response relationship up to approximately 8500 SED, which leveled off between 8500 and 9500 SED and fell afterwards. The risk doubled at a cumulative occupational UV dose of 6126 SED. This level was considerably lower compared to the recent analysis of the entire study population of 836 participants suffering from BCC at commonly sun-exposed and non-sun-exposed areas of the skin, where the risk to develop BCC doubled at 7945 SED [10]. The lower doubling dose in the present analysis is explained by the additional adjustment for sex, age, age^2^, Fitzpatrick phototype and non-occupational UV exposure and the restriction to BCC cases in commonly sun-exposed body sites. This result is consistent with the higher BCC risk estimates for the current analysis in 643 out of 836 cases.

The non-occupational UV dose-response curve showed a weaker rise and a broader plateau not reaching a risk-doubling dose. Our findings are in accordance with the literature, where comparable dose-response curves for BCC risk and total hours of sun exposure were identified in several studies [13, 34, 35]. It was speculated that subgroup effects introduced a complex dose-response relationship and thereby possibly a decreasing BCC risk at high doses [34]. Poor tanners (never tan or more often burn than tan) compared to good tanners exhibited different risk profiles. While poor tanners showed a plateau curve as mentioned above, good tanners seemed to have a steadily increasing risk to develop BCC [13].

In the present analysis of 643 cases with incident BCC in commonly sun-exposed body sites and their controls yielded a doubled risk of disease occurrence for occupational, but not non-occupational UV exposure. We assume that the decisive point leading to risk doubling by occupational UV exposure is the acquisition of high UV doses in shorter time intervals during occupational outdoor work (e.g. 6000 SED in 15 years of outdoor work, [400 SED/year]) compared to the cumulative UV exposure during entire lifetime (e.g. 9100 SED private exposure in a 70-year old person [130 SED/year]). This assumption is supported by recent evidence of high occupational UV exposure levels in various outdoor occupations by Wittlich et al. [8, 36]

### Strengths and limitations

The strength of our study is based on the fact that we analyzed a population-based sample of participants with incident histologically confirmed BCC recruited from a nationwide dermatologist network and matched controls [10]. By excluding patients with recurrent BCC, we reduced possible bias introduced by behavior and exposure change after the diagnosis of skin cancer. The population-based study design ensures a high degree of representativeness for the general population in Germany and Central Europe compared to previous hospital-based cohorts [9].

It is difficult to quantify the exact amount of sun exposure without taking UV dose measurements. To ensure data quality and completeness the computer-based, standardized, well-elaborated interviews were performed by trained interviewers. By this, we overcame limitations of self-administered questionnaires like bias due to missing data. Moreover, we ensured that all individual life periods were taken into account.

We cannot entirely exclude detection bias introduced by different observers, because interviews concerning occupational and non-occupational UV exposure were not performed by the same investigator at the sites. However, physicians assessing non-occupational UV exposure, as well as experts of the German Social Accident Insurance Institutions performing occupational UV exposure assessments were trained beforehand, thereby decreasing the risk of detection bias.

To control for confounders, cases and controls were matched. Moreover, the multivariate regression analysis was adjusted for additional confounders. Unlike previously published research [9, 37], we assessed occupational and non-occupational UV exposure in detail during all periods of life using precise and valid instruments recently developed for this purpose [10, 36, 38]. However, due to the retrospective collection of the UV exposure data, recall bias cannot be excluded entirely due to the difficulty to remember past-time sun behavior and exposure.

To reduce recall bias, the participants were asked in the invitation letter to consider the major stages of their private and occupational life prior to the interview at the clinic, as described in detail in the methods section. Moreover, occupational UV exposure was assessed more precisely compared to previous studies by using insurance documents on the duration of outdoor occupations.

However, selection bias might have played a role within the control group, which had been offered a free dermatological full body skin check as an incentive for study participation. This offer could have influenced participants with preexisting suspicious lesions and sun worshippers worried about possible cancer risk to seek medical advice. As a result, an underestimation of the association of BCC risk and occupational and non-occupational UV exposure could have occurred. On the other hand, it cannot be completely excluded that the study especially attracted healthy controls, leading to an overestimation of the association of BCC risk and UV exposure (“healthy volunteer effect”).

This study demonstrates an effect of cumulative occupational UV exposures on a qualitative and quantitative level. However, in particular the absolute UV exposure values could be questioned. The contribution of high intermittent, childhood and juvenile UV exposures may have been underestimated and, together with a possible differential recall bias of occupational and non-occupational UV exposures the dose response relationship of BCC risk and UV exposure could have been influenced.

Despite the remaining limitations, we believe that our study confirms our previous report indicating that the development of BCC in outdoor workers is dependent on high occupational solar UV exposure [10].

## Conclusion

The risk to develop BCC due to high occupational UV exposure at capillitium, face, lip, neck, dorsum of the hands, forearms outside, and décolleté is doubled consistently, irrespective of phototype, tumor localization and histological subtype. Most commonly affected body sites were head and neck and the décolleté. There was a positive dose-response relationship of occupational UV exposure and BCC risk at capillitium, face, lip, neck, dorsum of the hands, forearms outside, décolleté. The BCC risk-doubling dose adjusted for age, age^2^, sex and non-occupational UV exposure was 6126 SED. Our study confirms our previous report that high occupational UV exposure in outdoor work is a relevant risk factor for BCC development.

## Supplementary information

**Additional file 1: Table S1.** Association of UV exposure and risk for BCC in cases with histopathological subtype nodular and matched controls (*n* = 403 in each group). **Table S2**. Association of UV exposure and risk for BCC in cases with histopathological subtype sclerodermiform and matched controls (*n* = 145 in each group). **Table S3**. Association of UV exposure and risk for BCC in cases with histopathological subtype superficial and matched controls (*n* = 28 in each group).

## Data Availability

The datasets generated and/or analysed during the current study are available on request.

## Abbreviations

BCC: Basal cell carcinoma
SED: Standard erythema dose
OR: Odds ratio
CI: Confidence interval
