# Supplementary material for: Basal cell carcinoma risk and solar UV exposure in occupationally relevant anatomic sites: do histological subtype, tumor localization and Fitzpatrick phototype play a role? A population-based case-control study
Source: J Occup Med Toxicol. 2020 Sep 10;15:28. doi: 10.1186/s12995-020-00279-8 (PMC7488106; doi:10.1186/s12995-020-00279-8)
Supplement: Supplementary file 1 — Additional file 1: Table S1. Association of UV exposure and risk for BCC in cases with histopathological subtype nodular and matched controls (n = 403 in each group). Table S2. Association of UV exposure and risk for BCC in cases with histopathological subtype sclerodermiform and matched controls (n = 145 in each group). Table S3. Association of UV exposure and risk for BCC in cases with histopathological subtype superficial and matched controls (n = 28 in each group). [file 12995_2020_279_MOESM1_ESM.docx]

**Supplementary material**

Table S1: Association of UV exposure and risk for BCC in cases with histopathological subtype nodular and matched controls (n = 403 in each group)

| **UV exposure** | **cases^a^** | **contr.^a^** | **OR^b^** | **(95% - CI)^b^** | **p** | **OR^c^** | **(95% - CI)^c^** | **p** |
| --- | --- | --- | --- | --- | --- | --- | --- | --- |
| **Total UV exposure (leisure time and occupational exposure)** | | | | | | | | |
| < 20th percentile  (< 8,985.0 SED) | 75 | 94 | Ref. | Ref. | Ref. | . | . | . |
| 20. - < 40th percentile  (8,985.0 – 10,632.6 SED) | 70 | 81 | 1.55 | 0.75 - 3.20 | 0.24 | . | . | . |
| 40th - < 60th percentile  (10,632.7 – 12,565.6 SED) | 82 | 78 | 1.65 | 0.84 - 3.23 | 0.146 | . | . | . |
| 60th - < 90th percentile  (12,565.7 – 18,006.7 SED) | 129 | 117 | 1.89 | 1.00 - 3.58- | 0.051 | . | . | . |
| ≥ 90th percentile  (≥ 18,006.8 SED) | 47 | 33 | 2.86 | 1.27 - 6.43 | 0.011 | . | . | . |
| **Total UV exposure (high versus moderate exposure)** | | | | | | | | |
| 40th - < 60th percentile  (10,632.7 – 12,565.6 SED) | 82 | 78 | Ref. | Ref. | Ref. | . | . | . |
| ≥ 90th percentile  (≥ 18,006.8 SED) | 47 | 33 | 1.73 | 0.92 - 3.27 | 0.088 | . | . | . |
| **Occupational UV exposure** | | | | | | | | |
| < 44th percentile  (< 2.9 SED) | 174 | 183 | Ref. | Ref. | Ref. | Ref. | Ref. | Ref. |
| 44th - < 60th percentile  (2.9 - 532.1 SED) | 51 | 67 | 1.06 | 0.61 - 1.83 | 0.842 | 1.06 | 0.61 - 1.84 | 0.831 |
| 60th - < 90th percentile  (532.2 - 5.870.4 SED) | 130 | 127 | 1.03 | 0.70 - 1.53 | 0.872 | 1.02 | 0.68 - 1.51 | 0.933 |
| ≥ 90th percentile  (≥ 5,870.5 SED) | 48 | 26 | 2.09 | 1.09 - 4.00 | 0.027 | 2.09 | 1.09 - 4.01 | 0.027 |
| **Occupational UV exposure (high versus moderate exposure)** | | | | | | | | |
| 44th - < 60th percentile  (2.9 - 532.1 SED) | 51 | 67 | Ref. | Ref. | Ref. | Ref. | Ref. | Ref. |
| ≥ 90th percentile  (≥ 5,870.5 SED) | 48 | 26 | 1.97 | 0.91 - 4.26 | 0.084 | 1.97 | 0.91 - 4.26 | 0.086 |

**^a^** number of cases and controls (contr.)

**^b^** adjusted for age, age^2^, sex, phototype

**^c^** adjusted for age, age², sex, phototype and non-occupational UV exposure

**Table S2: Association of UV exposure and risk for BCC in cases with histopathological subtype sclerodermiform** **and matched controls (n = 145 in each group)**

| **UV exposure** | **cases^a^** | **contr.^a^** | **OR^b^** | **(95% - CI)^b^** | **p** | **OR^c^** | **(95% - CI)^c^** | **p** |
| --- | --- | --- | --- | --- | --- | --- | --- | --- |
| **Total UV exposure (leisure time and occupational exposure)** | | | | | | | | |
| < 20th percentile  (< 8,985.0 SED) | 31 | 32 | Ref. | Ref. | Ref. | . | . | . |
| 20. - < 40th percentile  (8,985.0 - 10,632.6 SED) | 40 | 35 | 1.82 | 0.62 … 5.34 | 0.276 | . | . | . |
| 40th - < 60th percentile  (10,632.7 - 12,565.6 SED) | 33 | 32 | 1.56 | 0.56 … 4.33 | 0.396 | . | . | . |
| 60th - < 90th percentile  (12,565.7 - 18,006.7 SED) | 34 | 36 | 1.67 | 0.53 … 5.26 | 0.384 | . | . | . |
| ≥ 90th percentile  (≥ 18,006.8 SED) | 7 | 10 | 1.02 | 0.24 … 4.30 | 0.976 | . | . | . |
| **Total UV exposure (high versus moderate exposure)** | | | | | | | | |
| 40th - < 60th percentile  (10,632.7 - 12,565.6 SED) | 33 | 32 | Ref. | Ref. | Ref. | . | . | . |
| ≥ 90th percentile  (≥ 18,006..8 SED) | 7 | 10 | 0.66 | 0.19 … 2.27 | 0.506 | . | . | . |
| **Occupational UV exposure** | | | | | | | | |
| < 44th percentile  (< 2.9 SED) | 67 | 69 | Ref. | Ref. | Ref. | Ref. | Ref. | Ref. |
| 44th - < 60th percentile  (2.9 - 532.1 SED) | 23 | 29 | 0.79 | 0.36 … 1.74 | 0.563 | 0.67 | 0.29 … 1.53 | 0.344 |
| 60th - < 90th percentile  (532.2 - 5,870.4 SED) | 44 | 37 | 1.46 | 0.71 … 3.01 | 0.308 | 1.55 | 0.74 … 3.24 | 0.243 |
| ≥ 90th percentile  (≥ 5,870.5 SED) | 11 | 10 | 1.35 | 0.38 … 4.76 | 0.637 | 1.29 | 0.36 … 4.63 | 0.692 |
| **Occupational UV exposure (high versus moderate exposure)** | | | | | | | | |
| 44th - < 60th percentile  (2.9 - 532.1 SED) | 23 | 29 | Ref. | Ref. | Ref. | Ref. | Ref. | Ref. |
| ≥ 90th percentile  (≥ 5,870.5 SED) | 11 | 10 | 1.71 | 0.50 … 5,80 | 0.390 | 1.93 | 0.56 … 6.70 | 0.300 |

**^a^** number of cases and controls (contr.)

**^b^** adjusted for age, age^2^, sex, phototype

**^c^** adjusted for age, age^2^, sex, phototype and non-occupational UV exposure

Table S3: Association of UV exposure and risk for BCC in cases with histopathological subtype superficial and matched controls (n = 28 in each group)

| **UV exposure** | **cases^a^** | **contr.^a^** | **OR^b^** | **(95% - CI)^b^** | **p** | **OR^c^** | **(95% - CI)^c^** | **p** |
| --- | --- | --- | --- | --- | --- | --- | --- | --- |
| **Total UV exposure (leisure time and occupational exposure)** | | | | | | | | |
| < 20th percentile  (< 8,985.0 SED) | 1 | 6 | . | . | . | . | . | . |
| 20. - < 40th percentile  (8,985.0 – 10,632.6 SED) | 7 | 3 | . | . | . | . | . | . |
| 40th - < 60th percentile  (10,632.7 – 12,565.6 SED) | 10 | 8 | . | . | . | . | . | . |
| 60th - < 90th percentile  (12,565.7 – 18,006.7 SED) | 7 | 7 | . | . | . | . | . | . |
| ≥ 90th percentile  (≥ 18,006.8 SED) | 3 | 4 | . | . | . | . | . | . |
| **Total UV exposure (high versus moderate exposure)** | | | | | | | | |
| 40th - < 60th percentile  (10,632.7 – 12,565.6 SED) | 10 | 8 | . | . | . | . | . | . |
| ≥ 90th percentile  (≥ 18,006.8 SED) | 3 | 4 | . | . | . | . | . | . |
| **Occupational UV exposure** | | | | | | | | |
| < 44th percentile  (< 2.9 SED) | 10 | 12 | Ref. | Ref. | Ref. | Ref. | Ref. | Ref. |
| 44th - < 60th percentile  (2.9 - 532.1 SED) | 4 | 5 | 1.65 | 0.17 … 16.30 | 0.666 | 1.45 | 0.13 … 15.76 | 0.761 |
| 60th - < 90th percentile  (532.2 - 5.870.4 SED) | 9 | 9 | 1.67 | 0.22 … 12.69 | 0.622 | 1.58 | 0.21 … 12.15 | 0.658 |
| ≥ 90th percentile  (≥ 5,870.5 SED) | 5 | 2 | 2.84 | 0.18 … 43.96 | 0.456 | 2.73 | 0.18 … 42.48 | 0.472 |
| **Occupational UV exposure (high versus moderate exposure)** | | | | | | | | |
| 44th - < 60th percentile  (2.9 - 532.1 SED) | 4 | 5 | Ref. | Ref. | Ref. | Ref. | Ref. | Ref. |
| ≥ 90th percentile  (≥ 5,870.5 SED) | 5 | 2 | 1.71 | 0.11 … 26.01 | 0.698 | 1.89 | 0.12 … 29.81 | 0.652 |

**^a^** number of cases and controls (contr.)

**^b^** adjusted for age, age^2^, sex, phototype

**^c^** adjusted for age, age², sex, phototype and non-occupational UV exposure
